# Prokaryotic Argonaute Protein from Natronobacterium gregoryi Requires RNAs To Activate for DNA Interference *In Vivo*

**DOI:** 10.1128/mbio.03656-21

**Published:** 2022-03-28

**Authors:** Jiani Xing, Lixia Ma, Xinzhen Cheng, Jinrong Ma, Ruyu Wang, Kun Xu, Joe S. Mymryk, Zhiying Zhang

**Affiliations:** a Key Laboratory of Animal Genetics, Breeding and Reproduction of Shaanxi Province, College of Animal Science and Technology, Northwest A&F University, Yangling, Shaanxi, China; b Changzhi Medical College, Changzhi, Shanxi, China; c Department of Microbiology & Immunology, Oncology and Otolaryngology, The University of Western Ontario, London, Ontario, Canada; Duke University School of Medicine

**Keywords:** NgAgo, *in vivo*, DNA interference, activation, bacteriophage T7

## Abstract

The Argonaute proteins are present in all three domains of life, which are archaea, bacteria, and eukarya. Unlike the eukaryotic Argonaute proteins, which use small RNA guides to target mRNAs, some prokaryotic Argonaute proteins (pAgos) use a small DNA guide to interfere with DNA and/or RNA targets. However, the mechanisms of pAgo natural function remain unknown. Here, we investigate the mechanism by which pAgo from Natronobacterium gregoryi (NgAgo) targets plasmid and bacteriophage T7 DNA using a heterologous Escherichia coli-based model system. We show that NgAgo expressed from a plasmid linearizes its expression vector. Cotransformation assays demonstrate that NgAgo requires an RNA in *trans* that is transcribed from the bacteriophage T7 promoter to activate cleavage of a cotransformed plasmid, reminiscent of the *trans*-RNA function in CRISPR/Cas9. We propose a mechanism to explain how NgAgo eliminates invading foreign DNA and bacteriophage. By leveraging this discovery, we show that NgAgo can be programmed to target a plasmid or a chromosome locus.

## INTRODUCTION

Argonaute proteins are a highly diverse family of guide-dependent nucleases found in all three domains of life (1–3). They were first discovered in eukaryotes as a key player in the RNA interference system, where they use small RNA guides to locate mRNA targets for regulating gene expression and suppressing mobile genetic elements (4, 5). Homologous prokaryotic Argonaute proteins (pAgos) have also been found in bacteria and archaea but are extremely diverse compared with eukaryotic Argonaute proteins (1–3). Unlike their eukaryotic counterparts, some pAgos have been shown to use small single-stranded DNA (ssDNA) as guides to act *in vitro* as nucleases with distinct specificity toward DNA targets (6–12). Others, such as MpAgo, use a 5′-OH RNA as a guide sequence to target RNA or single-stranded DNA sequences (13), while RsAgo utilizes RNA to target a DNA sequence (14). However, the mechanism of guide DNA generation, DNA target discrimination, and the natural function of pAgos *in vivo* remains, in general, unknown. Swarts et al. (15) previously proposed a DNA chopping mechanism for small DNA guide generation. They showed that *Thermus thermophiles* Argonaute (TtAgo) and Methanocaldococcus jannaschii Argonaute (MjAgo) proteins exhibited a guide-independent nuclease activity, coined as “chopping activity” (11, 15). This chopping activity exhibits two characteristics: most chopping substrates are derived from foreign invading plasmids or bacteriophages, and the chopping activity is extremely low. By chopping, pAgos first nonspecifically cut invading plasmids or bacteriophages, generating short fragments of foreign DNA, which are subsequently loaded on Argonaute. By an unknown mechanism, Argonaute protein then can release one strand of DNA while retaining the other strand as the guide DNA. Kuzmenko et al. reported that Clostridium butyricum Argonaute (CbAgo) showed a guide DNA loading preference at multicopy genetic loci within its host genome (10). They also found that loading of CbAgo with locus-specific small DNA guides depends on both its intrinsic endonuclease activity and the cellular double-strand break repair machinery. However, the natural functions of pAgos loaded with guide DNA remain unknown.

Many natural hosts expressing pAgos live in harsh and extreme environments, making it difficult to characterize pAgo function in homologous host cells. In addition, most pAgos are expressed poorly at 37°C in Escherichia coli BL21 and its derivatives (6, 7, 9, 10, 13, 16). Consequently, most published pAgo research is focused on protein structure and enzyme activity using *in vitro* cleavage assays (9, 11, 17–19), with limited data from *in vivo* studies (10, 20–24). As such, their *in vivo* functions remain elusive, with limited function characterization.

In the present study, we provide evidence that NgAgo heterologously expressed in E. coli BL21(DE3) cells linearizes its expression vector (pET28a based). Using cotransformation experiments, we determined that NgAgo requires *trans*-expressed RNAs from certain promoters to target a cotransformed plasmid, providing strong evidence that NgAgo-induced DNA interference requires RNA for activation *in vivo*. We also discovered that NgAgo expressed in E. coli BL21(DE3) can protect the bacterial cells from bacteriophage T7 infection, leading to significantly reduced PFU production and disrupted T7 genomic DNA. Based on our data, we propose a mechanism in which NgAgo interferes with foreign DNA in an RNA-activated manner. This discovery provides us with the tools to program NgAgo to target foreign nucleic acids, including both plasmid and genomic loci.

## RESULTS

### NgAgo linearizes its expression plasmid in E. coli.

**FIG 1:**
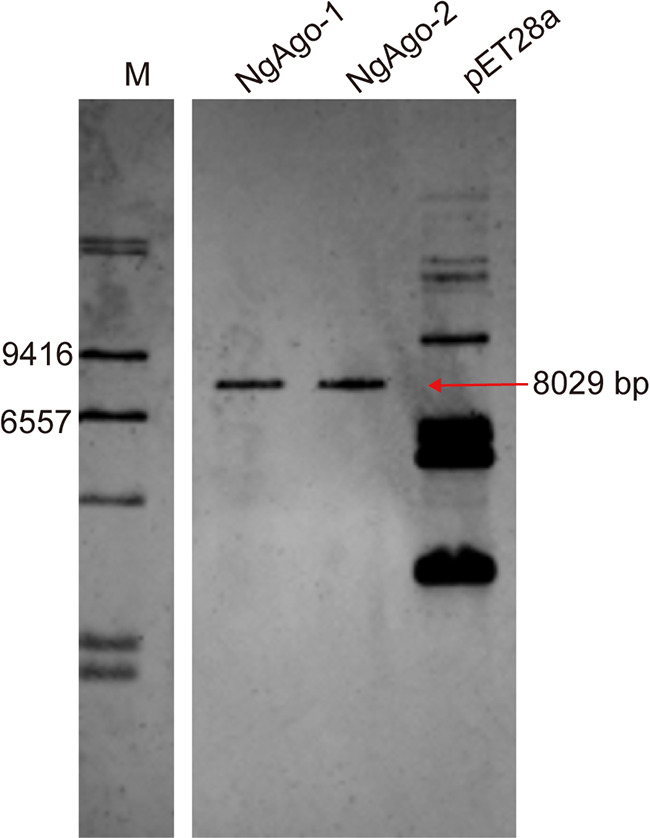
NgAgo linearizes its expression plasmid in E. coli. NgAgo-1/-2 represent two independent transformations with the pET28a-NgAgo plasmid. The parental pET28a plasmid represents the negative control. M, DNA molecular-weight marker II (11218590910; Roche). The arrow indicates the expected size of the linearized expression vector.

In our previous study (25), we showed that plasmid-based expression of either NgAgo or TtAgo (designated pET28a-NgAgo and pET28a-TtAgo) in E. coli BL21(DE3) resulted in rapid loss of their cognate expression vectors but had no adverse effect on the host cell genome. We hypothesized that this occurred via targeted endonuclease activity by NgAgo and TtAgo on their expression plasmids. To detect cleavage of the expression vectors, we used a digoxin-labeled probe corresponding to the kanamycin resistance gene (see Fig. S1 in the supplemental material) on the plasmid to perform Southern blots. As expected, extracts prepared from the negative-control BL21(DE3) cells transformed with the parental pET28a plasmid (Fig. S2) displayed multiple differentially migrating bands consistent with various DNA isoforms, representing supercoiled, nicked, and linear forms of the plasmid. In stark contrast, two independent transformations of cells induced with isopropyl-β-d-thiogalactopyranoside (IPTG) to express NgAgo display a single band corresponding to the linearized vector (pET28a-NgAgo) (Fig. 1, NgAgo-1 and -2). Thus, expression of NgAgo in BL21(DE3) leads to the linearization of the pET28a-based expression plasmid in such a way that it cannot be recircularized and therefore is unable to replicate within cells. These data are also entirely consistent with our previous result that induction of pAgos (NgAgo, TtAgo, and RsAgo) expression induced the elimination of their expression plasmids (25).

10.1128/mbio.03656-21.3 FIG S1 Agarose gel electrophoresis characterization of probe for southern blotting. Lane 1 is amplified PCR product of kanamycin probe using Kan-F and Kan-R. Lane 2 is Digoxin labeled probe. M is DL2000 marker. Download FIG S1, TIF file, 1.0 MB. Copyright © 2022 Xing et al. 2022 Xing et al. https://creativecommons.org/licenses/by/4.0/ This content is distributed under the terms of the Creative Commons Attribution 4.0 International license.

10.1128/mbio.03656-21.4 FIG S2 Schematic diagram of plasmid pET28a (A) and pUC57 (B). Download FIG S2, TIF file, 1.2 MB. Copyright © 2022 Xing et al. 2022 Xing et al. https://creativecommons.org/licenses/by/4.0/ This content is distributed under the terms of the Creative Commons Attribution 4.0 International license.

### Effect of NgAgo expression on cotransformed plasmids.

After determining that expression of NgAgo caused linearization of its expression vector, we then asked whether NgAgo expression can also linearize a second cotransformed plasmid. To address this question, we chose two other commonly used cloning and expression plasmids, pUC57 (Fig. S2) and pET32a, since both plasmids confer ampicillin resistance, an alternative selectable marker.

**FIG 2:**
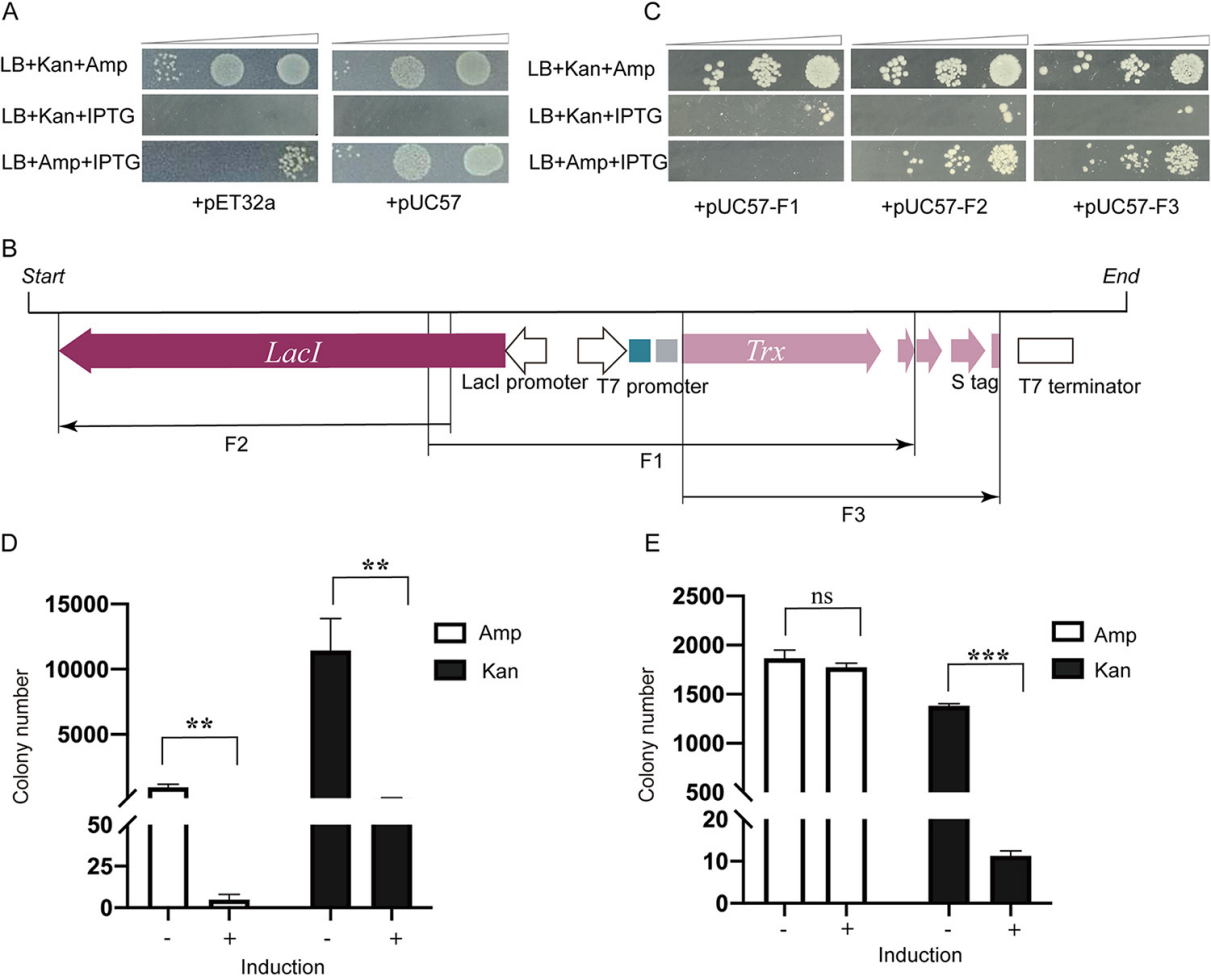
Effect of NgAgo expression on cotransformed plasmids. (A) Spot assay of pET28a-NgAgo and pET32a (left) or pUC57 (right) cotransformation. Serial dilutions of transformed cells were plated and grown on the indicated medium. (B) Schematic diagram of fragments F1, F2, and F3 derived from pET32a. (C) Spot assay of pET28a-NgAgo and pUC57-F1 (left)/pUC57-F2 (middle)/pUC57-F3 (right) cotransformation. Serial dilutions of transformed cells were plated and grown on the indicated medium. (D) Colony number measured from retransformation of pET28a-NgAgo (Kan) and pUC57-F1 (Amp) with and without IPTG induction. (E) Colony number measured by retransformation of pET28a-NgAgo (Kan) and pUC57 (Amp), with and without IPTG induction. Means and standard deviations (SD) from 3 independent biological replicates are shown for panels D and E. Error bars represent the means ± SD.

BL21(DE3) cells transformed with combinations of pET28a-NgAgo and either pET32a or pUC57 grew well as visible colonies on LB+Kan+Amp plates in the absence of IPTG induction, demonstrating that they can cosurvive and replicate within the same host cell without interference (Fig. 2A, top). We selected cells cotransformed with pET28a-NgAgo and either pET32a or pUC57 and performed a spot plating assay in which serial dilutions of cells were plated with or without IPTG to induce NgAgo expression (Fig. 2A). Consistent with the observations obtained in Fig. 1 and our previous report (25), NgAgo induction with IPTG led to the elimination of its own expression plasmid, as no visible colony growth was observed on the LB+Kan+IPTG plates (Fig. 2A, middle). LB+Amp+IPTG plates were used to examine the effect of NgAgo expression on the cotransformed pET32a or pUC57 plasmid. Surprisingly, the expression of NgAgo resulted in the elimination of the cotransformed plasmid pET32a, based on a large decrease in ampicillin-resistant colonies compared to that observed on the LB+Kan+Amp plate (Fig. 2A, left and bottom). In stark contrast, induction of NgAgo expression had no effect on the number of ampicillin-resistant colonies in cells cotransformed with pUC57 (Fig. 2A, right and bottom). NgAgo expression from the expression vector also had no adverse effect on the host cell genome, given that there’s minor difference of colony number on LB plates with or without IPTG induction (Fig. S3). This result suggested that pET32a, but not pUC57, contained a sequence targeted by NgAgo.

10.1128/mbio.03656-21.5 FIG S3 pET28a-NgAgo has no adverse effect on the bacterial genome but specifically eliminated plasmid pET32a after IPTG induction. pET28a-NgAgo was cotransformed with pET32a, and the same amount of bacterial suspension was plated on Amp plates (A), Kan plates (B), and LB plates with no antibiotics (C); IPTG was added as indicated. pET28a-NgAgo was cotransformed with pUC57 and the same amount of bacterial suspension was plated on Amp plates (D), Kan plates (E), and LB plates with no antibiotics (F); IPTG was added as indicated. The plates were photographed using agarose gel imaging system (JS-2012; Peiqing Science & Technology, Shanghai, China) with inverted color. Download FIG S3, TIF file, 1.1 MB. Copyright © 2022 Xing et al. 2022 Xing et al. https://creativecommons.org/licenses/by/4.0/ This content is distributed under the terms of the Creative Commons Attribution 4.0 International license.

To determine the essential region of pET32a necessary for NgAgo-mediated elimination, we divided it into three overlapping fragments, excluding the replication origin and the marker gene (Amp^r^) regions, and cloned these three individual fragments (F1, F2, and F3) into the nontargeted pUC57 plasmid. These were designated pUC57-F1, pUC57-F2, and pUC57-F3, as shown in Fig. 2B. We repeated the spot-plating assays using these derivatives to determine if any of these three regions could confer susceptibility to pET28a-NgAgo after induction (Fig. 2C). After cotransformation with pET28a-NgAgo into BL21(DE3), only the pUC57-F1 transformants exhibited a dramatic reduction in colony number similar to that from the cotransformed pET32a plasmid (Fig. 2C, left and bottom). This result suggests that the fragment F1 contains an essential sequence necessary for NgAgo to target the cotransformed plasmid.

To rule out any contribution related to artifacts of the cloning process, we generated a second clone containing the F1 fragment in the reverse orientation in the pUC57 vector, named pUC57-F1(r). Like pUC57-F1, the plasmid with the inverted F1 fragment was similarly sensitive to NgAgo, ruling out confounding effects from sequence orientation or cloning junctions (data not shown).

To further confirm the observations that the cotransformed pUC57-F1 plasmid was targeted by NgAgo for elimination, colony number was measured after retransformation of plasmid DNA isolated from transformed BL21(DE3) cells with or without IPTG induction. DNA from the pUC57 cotransformations was used as a control. The retransformation assay also indicated that NgAgo expression significantly reduced the content of pUC57-F1 plasmid (Fig. 2D) compared with the control group pUC57 in IPTG-induced cells (Fig. 2E). This result suggests that the F1 fragment derived from pET32a can trigger NgAgo to target a cotransformed plasmid in *trans*.

### The bacteriophage T7 promoter sequence on a cotransformed plasmid is essential for triggering NgAgo targeting.

**FIG 3:**
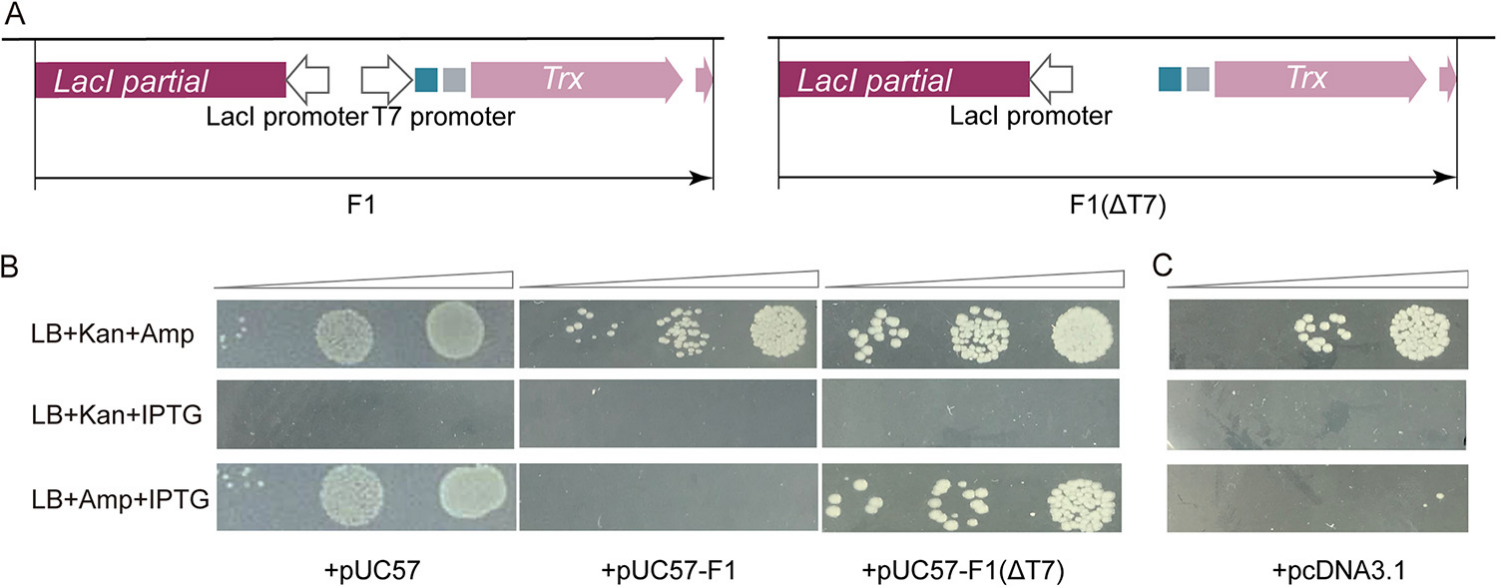
Bacteriophage T7 promoter sequence on a cotransformed plasmid plays an essential role for NgAgo targeting. (A) Schematic diagram of fragments F1 and F1(ΔT7). (B) Spot plating assay of pET28a-NgAgo and pUC57, pUC57-F1, or pUC57-F1(ΔT7) cotransformation. (C) Spot plating assay of pET28a-NgAgo and pcDNA3.1 cotransformation.

The pET32a-derived fragment F1 corresponds to 728 bp containing the bacteriophage T7 promoter as well as sequences for expression of foreign proteins, including the thioredoxin fusion gene (TrxA), a thrombin cleavage site, S-tag, and enterokinase cleavage site (Fig. 3A). To pinpoint the crucial sequence within this fragment, we first deleted the T7 promoter sequence [designated pUC57-F1(ΔT7)] and examined its sensitivity to NgAgo targeting using the spot-plating assay. As shown in Fig. 3B, pUC57-F1(ΔT7) was no longer targeted by NgAgo, suggesting that the bacteriophage T7 promoter plays an essential role in triggering NgAgo to target the cotransformed pUC57-F1 plasmid. This result prompted us to ask whether the T7 promoter DNA sequence itself or T7 promoter-driven expression of downstream polypeptides plays a role in inducing NgAgo to target the cotransformed plasmid. To address this question, we tested another T7 promoter-containing plasmid, pcDNA3.1, which does not share any sequence similarity downstream of the T7 promoter with the plasmid pET32a. Figure 3C clearly demonstrated that cotransformed pcDNA3.1 was also targeted by NgAgo, similar to pUC57-F1. This rules out a role for any specific polypeptides downstream of the T7 promoter in inducing NgAgo to target the cotransformed plasmid.

Given the fact that the spot-plating assays were conducted in BL21(DE3) strain and that NgAgo protein expression also employs a bacteriophage T7 polymerase-based system, we asked whether T7 polymerase physically interacts with NgAgo protein recruiting the nuclease to the T7 promoter sequence site for vector cleavage. To test this hypothesis, we performed coimmunoprecipitation assays but failed to detect any physical interaction between the two proteins (data not shown). No interaction was detected using yeast two-hybrid tests (data not shown). These results suggest that the bacteriophage T7 polymerase is unlikely to physically interact with the NgAgo protein.

### Bacteriophage T7 promoter-driven transcripts induce NgAgo to target the cotransformed plasmid DNA.

**FIG 4:**
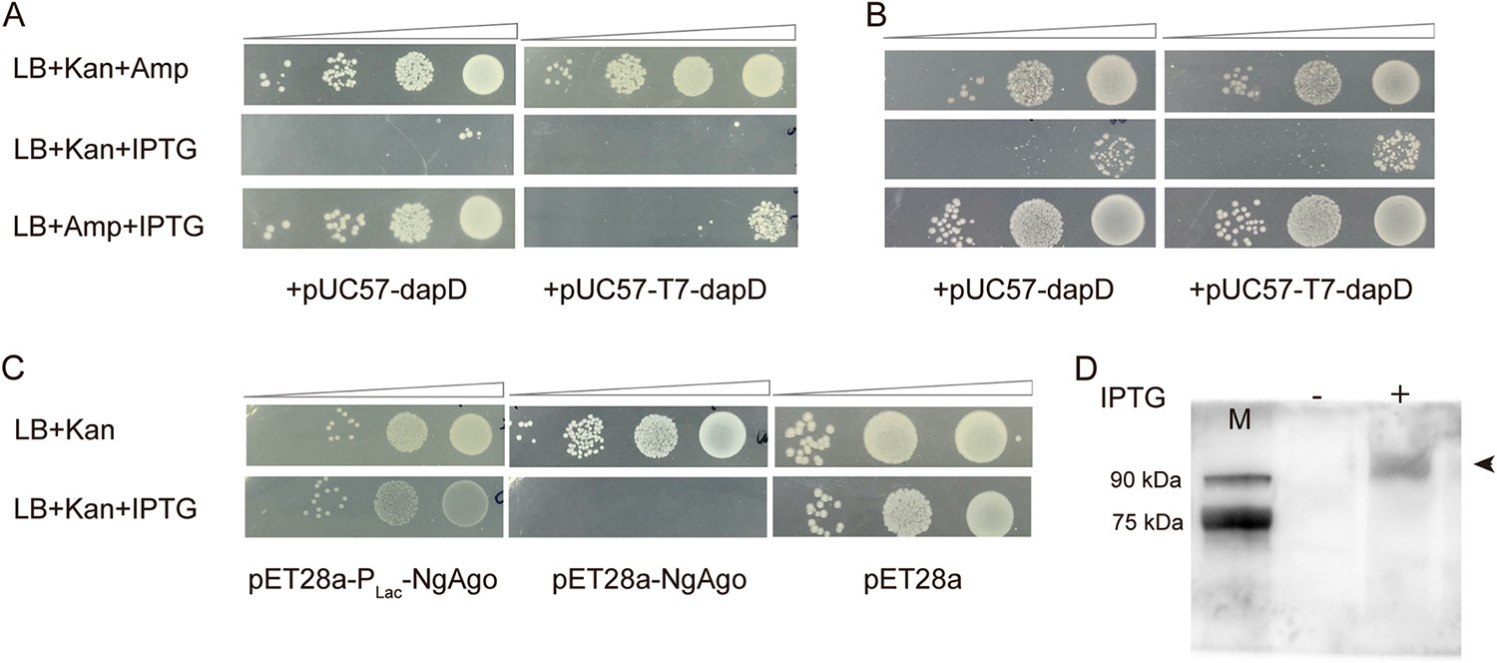
Bacteriophage T7 promoter-driven transcript induces NgAgo to target the cotransformed plasmid DNA. (A) Spot plating assay of pET28a-P_Tac_-NgAgo and pUC57-dapD, pUC57-T7-dapD cotransformation in BL21(DE3). (B) Spot plating assay of pET28a-P_Tac_-NgAgo and pUC57-dapD, pUC57-T7-dapD cotransformation in DH5α. (C) A comparison of the ability of NgAgo protein expressed from the Lac promoter and T7 promoter on expression vector plasmid elimination. (D) NgAgo protein expression by pET28a-P_Lac_-NgAgo was examined by Western blotting. IPTG was added as indicated. M, Easysee Western marker (DM201; TransGen Biotech, Beijing, China).

We next shifted our focus to a possible role of the T7 promoter-driven transcript. By comparing the sequences downstream of the T7 promoter between pET32a and pcDNA3.1, we did not find any sequence similarity, implying that a specific RNA directed by the T7 promoter is not required for NgAgo targeting of the cotransformed plasmid. We hypothesized that NgAgo nuclease activity could simply require any RNA transcript produced by the T7 promoter to activate specific targeting of the cotransformed template plasmid. To test the hypothesis, we constructed a pUC57-based plasmid with the T7 promoter driving 200 bp of the bacterial *dapD* gene sequence and conducted the cotransformation assays in two different bacterial strains, BL21(DE3) (Fig. 4A) and DH5α (Fig. 4B). Because the DH5α strain does not encode bacteriophage T7 RNA polymerase, we replaced the T7 promoter in the NgAgo expression vector with the P_Tac_ promoter, designated pET28a-P_Tac_-NgAgo. While the Tac promoter is also IPTG inducible, it is not dependent on T7 RNA polymerase and will function in both BL21(DE3) and DH5α cells.

As shown in Fig. 4A, the T7 promoter-containing cotransformed plasmid, pUC57-T7-dapD, was eliminated by NgAgo expressed from pET28a-P_Tac_-NgAgo vector in cotransformed BL21(DE3) cells after IPTG induction. In contrast, the equivalent cotransformed plasmid without the T7 promoter (pUC57-dapD) was not eliminated by NgAgo. However, when repeated in E. coli DH5α, which does not express the bacteriophage T7 RNA polymerase and, thus, does not generate an RNA transcript from the T7 promoter in plasmid pUC57-T7-dapD, NgAgo lost the ability to eliminate the T7 promoter containing pUC57 plasmid (pUC57-T7-dapD) (Fig. 4B). This result strongly supports our hypothesis that NgAgo requires an RNA transcript from the T7 promoter to activate its DNA interference activity and subsequent plasmid elimination, and that the RNA needs only correspond to a sequence in the vector to be targeted.

This raises the question of how the bacteriophage T7 promoter-driven RNA can activate NgAgo for specific elimination of a cotransformed plasmid. As for RNA species, the plasmid pUC57 normally produces several types of RNAs during plasmid replication within cells, including RNAI and RNAII, transcribed from the replication origin (26), and ampicillin resistance gene mRNA. In addition, pUC57 plasmid also contains the Lac promoter (P_Lac_) driving expression of the *lacZ* gene. In contrast to the transcript driven by the T7 promoter, these noncoding and coding RNAs produced by the plasmid pUC57 did not have the ability to activate NgAgo nuclease to cleave the pUC57. Interestingly, almost all pAgos isolated from either their homologous or heterologous cells exist as either a single-stranded DNA (ssDNA) or an RNA binding complex. The ssDNA or RNA sequences bound by pAgos mapped either to host genome sequence or plasmid and bacteriophage DNA sequence (6, 9, 24). These ssDNA or RNA-bound pAgo complexes apparently do not have nuclease activity to target the host genome or transformed plasmids. This suggests that they require an additional activation step, such as the presence of T7-driven RNAs, to achieve DNA interference via the bound ssDNAs and the complementary target DNA sequence.

Based on our results and previous reports, we hypothesized that these ssDNA-binding pAgo complexes require a special activating transcript, such as a T7 promoter-driven RNA, which is necessary for targeting DNA in a guide-dependent manner. This activation most likely happens in a sequence-complementary manner between NgAgo-binding ssDNA and the T7 promoter-derived RNA transcript, which we have termed an activation RNA (aRNA). After activation, the ssDNA binding NgAgo complex then specifically targets the plasmid by its ssDNA guide, or perhaps the aRNA also functions as a guide. In retrospect, this hypothesis aligns with our previous observation that expression of NgAgo and TtAgo in BL21(DE3) cells resulted in degradation of the expression plasmids, likely because the ssDNA binding NgAgo and TtAgo can be activated by their own transcript mRNA, which was driven by the T7 promoter in the pET28a vector. This results in linearization and loss of the expression plasmid after induction.

To test this model, we replaced the T7 promoter in pET28a-NgAgo with the Lac promoter (pET28a-P_Lac_-NgAgo). We chose the Lac promoter based on the observation that the transcript driven by the Lac promoter on plasmid pUC57 did not activate NgAgo to target the cotransformed plasmid pUC57 (Fig. 3B, left). Therefore, we assumed that Lac promoter-driven NgAgo transcript would not activate NgAgo to target its expression plasmid.

As expected, after being transformed into BL21(DE3) cells, the spot-plating assay showed that expression of the NgAgo protein driven by the Lac promoter (pET28a-P_Lac_-NgAgo) did not result in the elimination of the NgAgo expression vector, unlike what was observed when its expression was driven by the T7 promoter (Fig. 4C). A Western blot ruled out the possibility that the cloned promoters were nonfunctional, as NgAgo expression was detected from the vector containing the Lac promoter. We also verified Lac promoter activity by showing that it was sufficient to direct expression of eGFP (data not shown). Both assays confirmed that the Lac promoter works well in the cloned vector.

### Reconstitution of NgAgo expression by integration into heterologous BL21(DE3) host cells.

To further elucidate the mechanism by which pAgos might defend host cells from foreign DNA, we reconstituted their expression in BL21(DE3) cells by individually integrating the *NgAgo* gene or the *EGFP* gene into the BL21(DE) cell genome. This should mimic *NgAgo* gene expression in its natural host and help precisely define their function under endogenous expression. We designed an integration vector containing a T7 promoter-driven *NgAgo* gene expression cassette flanked by 400 bp of homologous chromosome integration sequence. To facilitate selection of integrated colonies, we incorporated a kanamycin resistance gene upstream of the *NgAgo* expression cassette. Based on a previous report (27), we targeted the integration vector into the BL21(DE3) genome at the coordinate of 1,300,270. With the aid of the Lambda Red recombination system and kanamycin selection, we obtained the *NgAgo* gene-integrated BL21(DE3) recombinant strain, named BL21::NgAgo. A control *EGFP* expression cassette was similarly integrated to create BL21::EGFP. We verified the integration site and *NgAgo* gene expression cassette sequence and protein expression (Fig. S4) and the green fluorescence expressed by BL21::EGFP strain after IPTG induction (Fig. S5).

10.1128/mbio.03656-21.6 FIG S4 Western blotting of NgAgo protein expressed via BL21(DE3) chromosomal integration in BL21(DE3) cells. IPTG was added as indicated, lane 1 was NgAgo expressed from integration vector pB-LA-Kan-T7-NgAgo-RA, and lane 2 is the negative control of lane 1. Lane 4 and lane 6 were two samples of NgAgo expressed from BL21(DE3) chromosome. Lane 3 and lane 5 were negative control of lane 4 and lane 6. M-Easysee Western marker was used (DM201; TransGen Biotech, Beijing, China). Download FIG S4, TIF file, 0.6 MB. Copyright © 2022 Xing et al. 2022 Xing et al. https://creativecommons.org/licenses/by/4.0/ This content is distributed under the terms of the Creative Commons Attribution 4.0 International license.

10.1128/mbio.03656-21.7 FIG S5 Visualization of BL21::EGFP colonies under ultraviolet light. Panels A and B are from two different trial attempts. Download FIG S5, TIF file, 2.5 MB. Copyright © 2022 Xing et al. 2022 Xing et al. https://creativecommons.org/licenses/by/4.0/ This content is distributed under the terms of the Creative Commons Attribution 4.0 International license.

**FIG 5:**
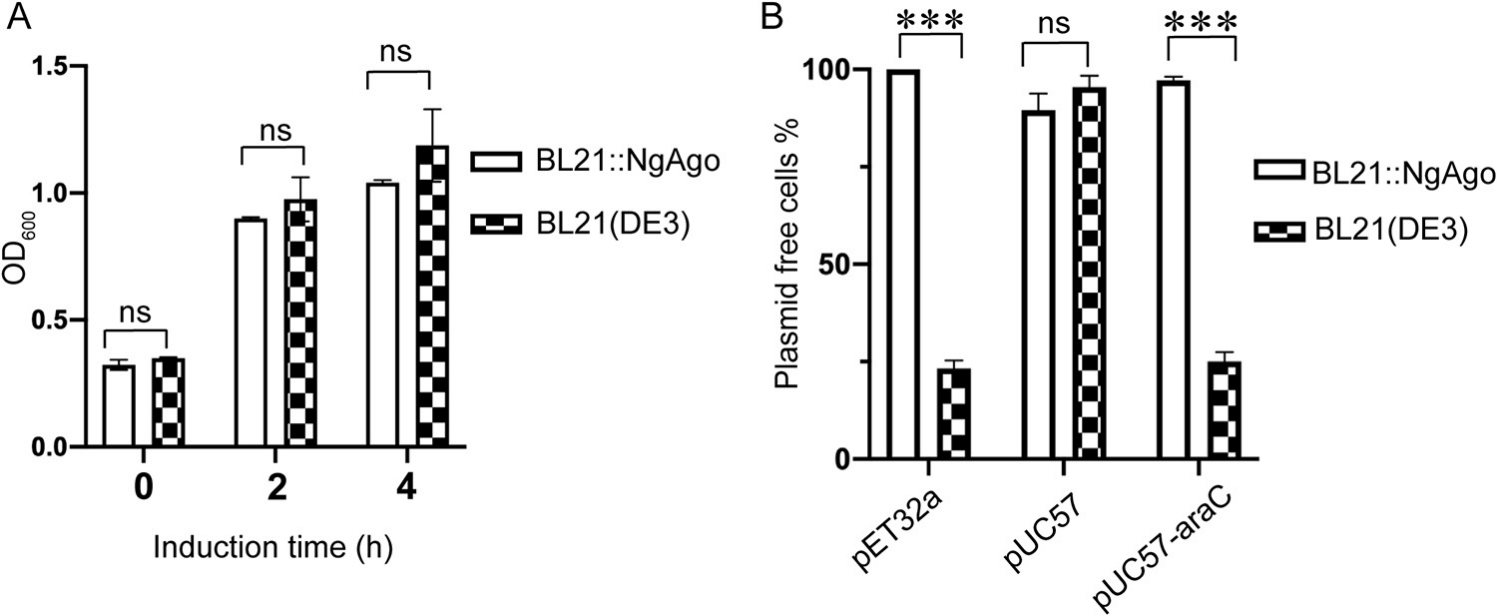
Reconstitution of NgAgo expression by integration into heterologous BL21(DE3) host cells. (A) OD_600_ values of NgAgo-integrated BL21(DE3) and wild-type BL21(DE3) at different time points after IPTG induction. (B) Comparison of plasmid maintenance efficiency between NgAgo-integrated BL21(DE3) and wild-type BL21(DE3). Means and standard deviations from 3 independent biological replicates are shown for panels A and B. Error bars represent the means ± SD. ns, not significant. ***, *P < *0.001. Data were analyzed by SPSS using a one-way ANOVA and charts were generated with Graph Prism 8.

We first examined whether expression of the endogenously integrated *NgAgo* gene affected bacterial cell growth by comparing optical density at 600 nm (OD_600_) values between BL21::NgAgo and BL21(DE3) at different time points after IPTG induction. Unlike what we observed for plasmid-based expression, induction of *NgAgo* expression from the integrated genomic copy did not affect BL21(DE3) cell growth (Fig. 5A).

We next tested whether endogenously expressed NgAgo can interfere with transformed plasmid DNA, similar to NgAgo expressed from the pET28a vector, to reduce plasmid maintenance efficiency, similar to a CbAgo-expressing strain (10). To perform these experiments, we constructed a pUC57-araC plasmid and transformed it in parallel with pET32a and pUC57 plasmids into BL21::NgAgo and BL21(DE3). Upon induction of NgAgo, we measured the efficiency of plasmid maintenance (Fig. 5B). The number of pET32a and pUC57-araC plasmid-free cells in BL21::NgAgo is significantly higher than that in the BL21(DE3) group after the fourth passage in LB medium supplemented with IPTG. In contrast, the BL21::NgAgo strain maintained the pUC57 plasmid until the seventh passage in LB medium supplemented with IPTG (Fig. S6). This result confirms that endogenously expressed NgAgo could rapidly eliminate those plasmids that generated the transcripts driven by the T7 or *araC* promoters, consistent with the result from CbAgo-expressing strains (10).

10.1128/mbio.03656-21.8 FIG S6 Plasmids maintenance assay for pET32a, pUC57-araC and pUC57 plasmids in BL21::NgAgo and BL21(DE3). (A) pET32a plasmid maintenance of passage 1, 2, and 4 in BL21::NgAgo and BL21(DE3). (B) pUC57-araC plasmid maintenance of passage 1, 2, and 4 in BL21::NgAgo and BL21(DE3). (C) pUC57 plasmid maintenance of passages 1, 2, and 7 in BL21::NgAgo and BL21(DE3). Means and standard deviations from 3 independent biological replicates are shown for panels A, B, and C. Error bars represent the means ± SD. ns, not significant; **, *P < *0.05; ***, *P < *0.001. Data were analyzed by SPSS using one-way ANOVA and graphs were generated with Graph Prism 8. Download FIG S6, TIF file, 1.1 MB. Copyright © 2022 Xing et al. 2022 Xing et al. https://creativecommons.org/licenses/by/4.0/ This content is distributed under the terms of the Creative Commons Attribution 4.0 International license.

### NgAgo protects BL21(DE3) cells from bacteriophage T7 infection by interfering with the integrity of phage genomic DNA.

Encouraged by the discovery that NgAgo requires T7 promoter-directed transcripts to activate its guide-dependent effects on foreign DNA in our experiments described above, we hypothesized that NgAgo should defend heterologous BL21(DE3) host cells against bacteriophage T7 infection, as this bacteriophage produces several T7-driven transcripts. To test this, we transformed pET28a-NgAgo plasmid into BL21(DE3) cells and challenged them with T7 phage.

**FIG 6:**
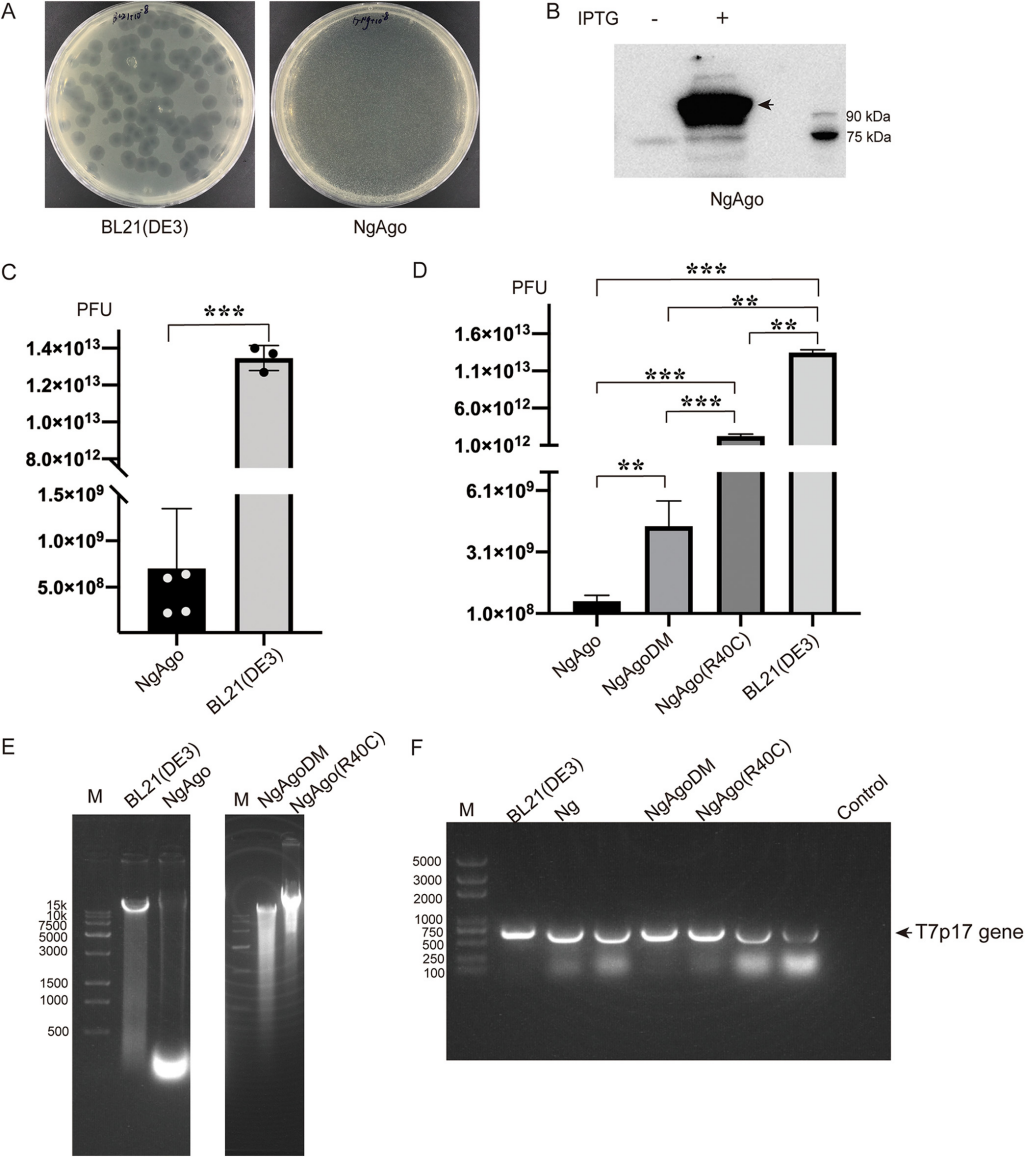
NgAgo protects BL21(DE3) cells from bacteriophage T7 infection by interfering with the integrity of phage genomic DNA. (A) Plaques of bacteriophage T7 on BL21(DE3) and NgAgo-expressing BL21(DE3). (B) NgAgo protein expression detected by Western blotting. (C) T7 PFU comparison of phage yield from infected NgAgo expressing BL21(DE3) and parental BL21(DE3). (D) T7 PFU yield comparison among infected NgAgo and NgAgo mutants in BL21(DE3). (E) T7 bacteriophage genomic DNA from infected wild-type BL21(DE3) and derivatives expressing NgAgo and NgAgo mutants. M, *Trans*15K DNA marker (TransGen Biotech, Beijing, China). (F) PCR amplification of the T7 p17 gene using phage genomic DNA from panel E as templates. Genomic DNA of BL21(DE3) was used as the control. The unmarked lanes were other samples unrelated to this article. M, *Trans*2K plus DNA marker (TransGen Biotech, Beijing, China). Means and standard deviations from 3 independent biological replicates are shown for panels C, D, and E. Error bars represent the means ± SD. *, *P < *0.05; **, *P < *0.01; ***, *P < *0.001. Data were analyzed by SPSS by one-way analysis of variance including least significant difference and Duncan multiple comparisons, and graphs were generated with Graph Prism 8.

Bacteriophage T7 efficiently infected BL21(DE3) cells, causing bacterial cells lysis and plaque formation. However, the BL21(DE3) cells expressing NgAgo were resistant to bacteriophage T7 infection, as no visible plaques was detected after infection with the same titer of T7 phage (Fig. 6A). NgAgo protein was expressed as determined by Western blotting (Fig. 6B). We concluded that NgAgo protein protected the heterologous host BL21(DE3) cells from bacteriophage T7 infection. An examination of the number of PFU of bacteriophage T7 produced from NgAgo transformation groups and the BL21(DE3) control group confirmed that NgAgo expression significantly decreased the relative titer of virus produced upon infection (Fig. 6C). Compared with the control BL21(DE3) cells, NgAgo expression dramatically reduced the number of PFU of progeny phage by more than 5 orders of magnitude.

To further study their host defense function, we tested several previously studied NgAgo mutants (6, 22) for their ability to protect from bacteriophage T7 infection (Fig. 6D). We generated and tested two mutants of NgAgo: (i) the double mutant NgAgoDM (D663A, D738A), which targets the DEDX active center of the NgAgo protein, constructed based on structural similarity with TtAgo and determined to be catalytically important in previous *in vitro* nuclease activity studies (6), and (ii) NgAgo (R40C), a single point mutation constructed and verified using the same method described in our previous *in vivo* loss-of-function mutation screen (25). Although NgAgoDM showed a decrease in protection compared with wild-type NgAgo, reducing PFU production by 1 order of magnitude, it still retained significant activity compared with control BL21(DE3) cells. This suggests that mutations within the putative enzyme active center do not completely abrogate NgAgo’s ability to protect against virus infection (Fig. 6D). The single point mutation NgAgo (R40C) was more significantly impaired but still able to reduce phage yield compared with the control BL21(DE3) cells. These results suggest that NgAgo employs a complicated mechanism for protection of the host cell from virus infection that relies on multiple different functions.

We also examined the integrity of T7 phage DNA extracted from infected cells by agarose gel electrophoresis. A DNA sample from the control BL21(DE3) cells displayed a sharp band of ∼40 kb, consistent with the full-length T7 phage genome size. However, DNA from NgAgo-expressing cells presented as a smear of DNA, indicative of degradation (Fig. 6E). Using these genomic DNA samples as the template, we confirmed that they represented T7 phage DNA by PCR amplification of the T7-encoded p17 gene (Fig. 6F). These data conclusively demonstrated that NgAgo expression protected E. coli from T7 phage infection by interfering with the integrity of phage genomic DNA.

## DISCUSSION

### Evidence that NgAgo nuclease activity requires RNA activation.

The pAgos have been long considered a microbe defense system against foreign DNA invasion and bacteriophage infection (28). However, the mechanism by which they function in the host cell remains to be determined. In the current study, we first demonstrated that plasmid-based expression of NgAgo in heterologous host BL21(DE3) cells caused linearization (Fig. 1) and loss of its cognate expression plasmid (Fig. 2A, middle). We assume that similar to observations that different sgRNAs generate different efficiencies of DNA cleavage in the CRISPR/Cas9 system, the ssDNAs bound to NgAgo could also possess a different efficiency in plasmid cleavage. Therefore, in a spot assay, with thousands of transformed cells in which each NgAgo protein presumably binds a different guide DNA, the remnant colonies in the spot assay might reflect those resulting from some of these less potent guide DNAs (Fig. 2A and C, middle). Nevertheless, our observation resolves the puzzle of why almost all pAgos, no matter their host source, are expressed poorly in BL21(DE3) cells with commonly used expression vectors at 37°C.

Using cotransformation experiments, we discovered that NgAgo can similarly target a second, cotransformed plasmid for elimination as measured by spot plating and plasmid recovery assays, presumably also by linearization (Fig. 2). Interestingly, not all cotransformed plasmids tested were eliminated, and detailed analysis revealed that the presence of a T7 promoter on the plasmid was necessary (Fig. 3) and that it had to be active to initiate targeting (Fig. 4). This discovery suggests that RNA products from the T7 promoter are a key component of the NgAgo for activation and/or target selection in this context.

### Proposed discriminator for NgAgo in host DNA and foreign DNA selection.

The mechanism by which pAgos differentiate between host DNA and foreign DNA remains unknown. A number of mechanisms have been proposed. The chromatin state of host strain genomic DNA may confer protection from cleavage by MjAgo, likely by bacterial chromatin proteins (11). Others have proposed that the AT/CG ratio is a marker for target selection (6), given that the heterologous sequence has lower GC content than those genes from the host genome (29). In addition, gene copy number may be another basis for pAgo target selection, since plasmid DNA usually possesses a higher copy number than genomic DNA, and the higher frequency of transcription likely induces the local unwinding of DNA helix structure, which may serve as the single-strand DNA structure for Argonaute protein targeting. Finally, a study of CbAgo (10) found that it cooperated with bacterial recombination protein RecBCD, which participates in the repair of double-strand breaks (DSBs) in bacterial cells, suggesting that the DNA replication frequency and the presence of the Chi (crossover hot spot instigator) sites is a possible mechanism for Argonaute recognition.

Although we have not discovered the exact reason why the transcripts driven by the T7 promoter can activate NgAgo nuclease activity in the current study, the T7 promoter, derived from T7 bacteriophage, has much stronger activity than the E. coli RNA polymerases (30). Given that the pAgos may act as a defense system and that copy number may be a determinant for Argonaute protein to distinguish self and non-self elements, we assume this may be a reason that makes the T7-driven transcript a target for Argonaute proteins.

### NgAgo protected a heterologous host from bacteriophage infection.

We also demonstrate that NgAgo can function in antiphage defense in heterologous E. coli BL21(DE3) host cells at 37°C (Fig. 6A and C). Transcripts driven by the promoter within bacteriophage T7 likely can activate NgAgo to target the phage in a guide-dependent manner, such that the expression of NgAgo protected heterologous host BL21(DE3) cells from phage infection. This finding suggests that as an ancient defense system, NgAgo can recapitulate this conserved function of Ago-mediated nucleic acid interference in the heterologous E. coli host. The *in vivo* functional activity of NgAgo against plasmids and phages in E. coli may not require its own endonuclease activity, as the inactive mutants NgAgoDM showed a significant, albeit smaller, effect compared to that of wild-type NgAgo on protection from T7 phage infection (Fig. 6D). Similar results were also reported with CbAgo in antiphage experiments (10). Indeed, many pAgos do not require cleavage of target DNA for their function, since many contain natural substitutions in their active sites that likely abrogate their endonuclease activity (9, 11, 14, 15). Nevertheless, future studies are warranted to more clearly elucidate the mechanisms responsible for pAgo *in vivo* activity in this readily amenable E. coli model system.

### The proposed mechanism for NgAgo function *in vivo*.

Mounting evidence has demonstrated that almost all pAgos isolated from either their homologous or heterologous host cells copurify with short single-stranded DNA or RNA (6, 9, 24), which suggested that these short binding DNAs or RNAs must somehow be involved in pAgo *in vivo* function. Swarts et al. proposed a random chopping model for pAgos to obtain these short DNA or RNA fragments (15). This model well explains why pAgos always coexist with short DNAs or RNAs. However, the majority of these pAgo-bound short single-stranded DNAs or RNAs are mapped to plasmids or bacteriophage sequences, while some of them are mapped to host genome (6, 24). Based on these observations, many researchers have tried to program the pAgos to target plasmids or genomic loci by providing a short single-stranded DNA or RNA as the guide but without success. Possibly, these nucleic acid-binding pAgos did not show any nuclease activity, as they need to be activated by RNAs expressed by foreign invading DNA, such as the RNAs directed by the T7 promoter. We assume that these RNAs have to be complementary with pAgo-binding ssDNA or guide DNA. In the current study, our data indicates that these single-stranded DNA- or RNA-bound pAgos do function in immune surveillance, with this additional activation step. Indeed, efficient cleavage activity requires a certain type of RNA, referred to here as aRNA, to activate pAgos for guide-dependent targeting.

**FIG 7:**
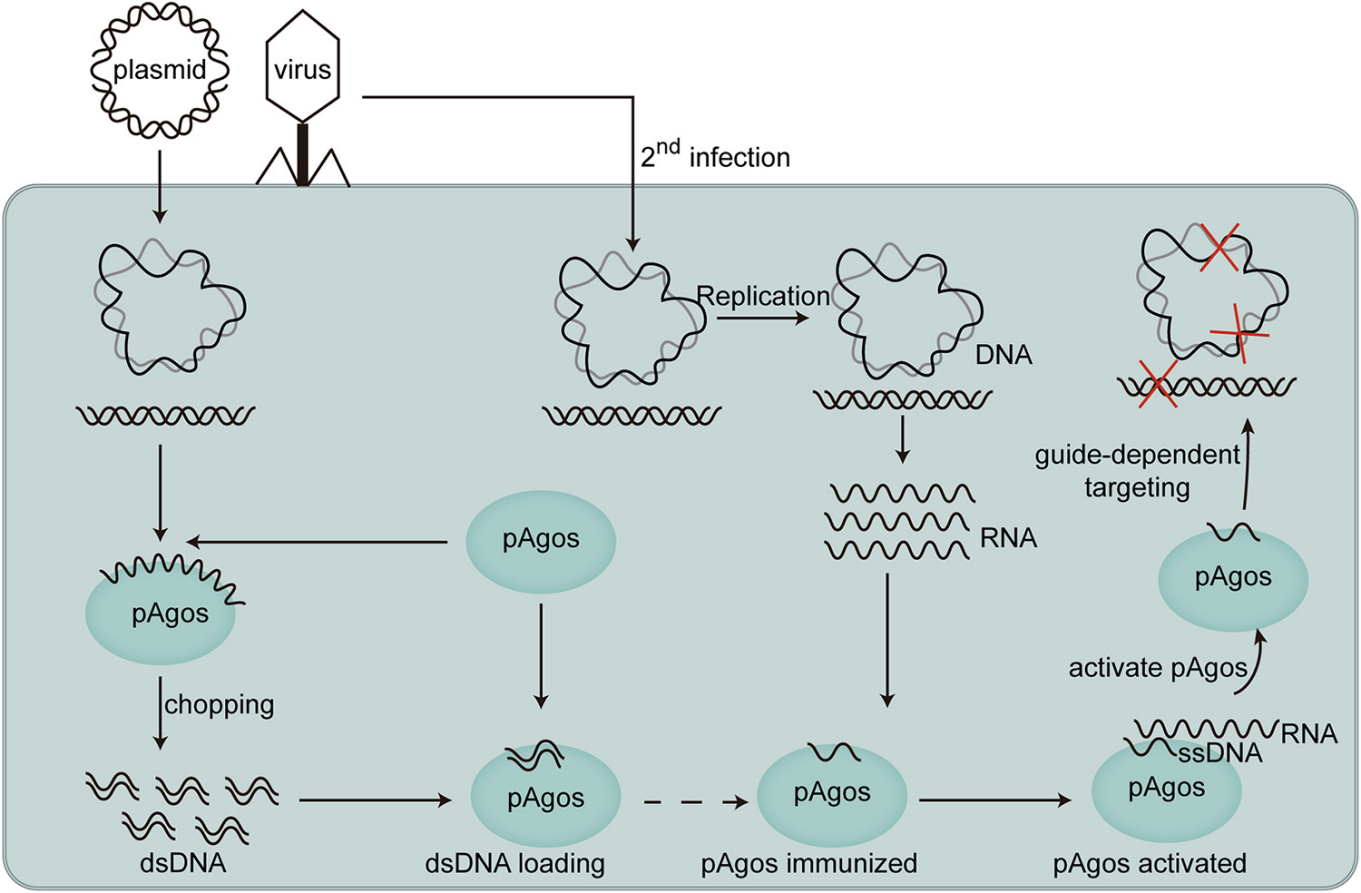
Proposed the mechanism by which pAgos function to defend host cells from foreign DNA or phage. Foreign DNA is nonspecifically generated and used to form immunized forms of pAgos that can be subsequently activated by foreign RNA to create guide specific targeting complexes.

Based on our observations combined with the previously reported research, we propose a novel mechanism by which pAgos defend their host cell against foreign DNA invasion and bacteriophage infection (Fig. 7). Starting with guide-independent nuclease activity/Argonaute chopping activity (15, 23), most substrates likely come from low-efficiency/nonspecific cleavage of invading plasmids or bacteriophages. This generates short fragments of foreign DNA that are loaded onto the pAgos. By an unknown mechanism, pAgos then can release one strand of DNA while retaining the other strand as guide DNA. We consider this chopping process the first step of the defense mechanism. In this first step, pAgos not only eliminate invading or infected viral DNA before they launch propagation but also initiate immunization by loading chopped small DNA from the foreign DNA onto pAgos. These immunized pAgos (guide-loaded Argonaute proteins) then function to confer immune surveillance. A mother cell might pass these DNA binding pAgos on to daughter cells. Whenever the same plasmid or bacteriophage again invades or infects the cell harboring these surveillance pAgos and launches replication, the aRNAs transcribed from these invading DNAs activate pAgo nuclease activity, likely in a DNA-RNA complementary manner. After nuclease activity is activated, the pAgos will target the invasive DNA with high efficiency in a guide-dependent manner, leading to complete elimination of invading plasmid or bacteriophage DNA (Fig. 7).

In summary, we demonstrate that NgAgo-induced DNA interference requires certain types of RNA for activation to target plasmids and can protect the BL21(DE3) bacterial cells from bacteriophage T7 infection. We proposed an *in vivo* working mechanism for pAgos that potentially can be used for genomics applications, particularly as an instrument to study genome architecture and DNA processing in prokaryotic and potentially eukaryotic systems.

## MATERIALS AND METHODS

### Strains and cultivation.

The E. coli BL21(DE3) and DH5α strains were used for electroporation and protein expression or vector construction, respectively, in this study. They were cultured in Luria-Bertani broth with the addition of the appropriate antibiotics or IPTG at 37°C. The final concentration of kanamycin was 50 μg/mL, ampicillin was 100 μg/mL, inducer l-arabinose (L8060; Solarbio, Beijing, China) was 10 mM, and inducer isopropyl β-d-thiogalactopyranoside (IPTG) was 0.5 mM for pET28a-based plasmid induction and 0.2 mM for T7-NgAgo/EGFP integrated BL21(DE3) strain induction.

### Plasmid construction.

The *NgAgo* (GenBank accession no. AFZ73749.1) was synthesized by ShineGene Molecular Biotech, Inc. (Shanghai, China), and cloned into pET28a.

The T7 promoter and fragments F1, F2, and F3 were amplified by PCR and inserted into the pUC57-simple vector. The constructed plasmids were named pUC57-F1/F2/F3. The T7 promoter sequence in F1 fragment was deleted by overlap PCR using primers F1-HindIII-F/NheI-T7_P del-R and F1-XhoI-R/NheI-T7_P del-F and replaced the F1 sequence in the pUC57-F1 plasmid. The constructed plasmid was named pUC57-F1(ΔT7). The F1-inverted sequence was amplified with primers F1-XhoI-F/F1-HindIII-R and replaced the F1 fragment in pUC57-F1, generating pUC57-F1(r).

The CAP binding site-P_Lac_-LacO sequence was amplified from pUC57-simple plasmid using primers BglII-CAP binding-F/XbaI-M13R-R and is referred to here as P_Lac_. The Tac promoter sequence was amplified from plasmid pGEX4T1 using primers BglII-P_Tac_-F/XbaI-P_Tac_-R and is referred to here as P_Tac_. The *araC*-araBAD promoter sequence was amplified from pKD46 vector using primers BglII-araC-R/XbaI-pBAD-R and is referred to here as pBAD. These different promoter sequences were cloned into pET28a-NgAgo between BglII and XbaI, generating the pET28a-P_Lac_/P_Tac_/pBAD-NgAgo plasmids.

The 200-bp-length *dapD* gene sequence was amplified from BL21(DE3) genomic DNA (accession number CP001509.3) using primers XhoI-dapD-F/HindIII-dapD-R and XhoI-T7-dapD-F/HindIII-dapD-R and inserted into the pUC57-simple vector between XhoI and HindIII, generating pUC57-dapD and pUC57-T7-dapD, respectively. The *araC* gene was amplified from pET28a plasmid using primers XhoI-araC-F/HindIII-araC-R and cloned into pUC57-simple vector between XhoI and HindIII, generating pUC57-araC.

Integration plasmids pB-LA-Kan-T7-NgAgo-RA and pB-LA-Kan-T7-EGFP-RA were constructed as follows. (i) Plasmids pET28a-NgAgo and pXL-BacII were digested with BglII and XhoI, and the T7-NgAgo fragment was inserted into pXL-BacII, generating the pB-NgAgo plasmid. (ii) The T7 terminator sequence was amplified from pET28a-NgAgo using primers T7-Term-F(XhoI)/T7-Term-F2(BglII-XhoI) and inserted into pB-NgAgo between XhoI and HindIII, generating pB-NgAgo-term. (iii) The kanamycin gene sequence was amplified from pET28a-NgAgo using primers Kan-F (NotI)/Kan-R(BamHI) and inserted into pB-NgAgo-term, generating pB-Kan-NgAgo-term. (iv) Left arm (LA) and right arm (RA) sequences were amplified from BL21(DE3) genomic DNA using primers LeftArm-F (BamHI)/LeftArm-R(NotI) and RighArm-F(KpnI)/RightArm-R(HindIII-BamHI) and ligated with pB-Kan-NgAgo-term, generating pB-LA-Kan-NgAgo-term-RA.

The *EGFP* gene sequence was amplified from pET32a-EGFP using primers NdeI-EGFP-F and XhoI-EGFP-R and cloned into pB-LA-Kan-NgAgo-term-RA between NdeI and XhoI, generating pB-LA-Kan-EGFP-term-RA.

The above-mentioned primers are listed in Table S2 in the supplemental material.

10.1128/mbio.03656-21.1 TABLE S1 Plasmids used in this study. Download Table S1, XLSX file, 0.01 MB. Copyright © 2022 Xing et al. 2022 Xing et al. https://creativecommons.org/licenses/by/4.0/ This content is distributed under the terms of the Creative Commons Attribution 4.0 International license.

10.1128/mbio.03656-21.2 TABLE S2 Primers used in this study. Download Table S2, XLSX file, 0.01 MB. Copyright © 2022 Xing et al. 2022 Xing et al. https://creativecommons.org/licenses/by/4.0/ This content is distributed under the terms of the Creative Commons Attribution 4.0 International license.

### NgAgo mutant construction.

Arginine 40 was replaced with cysteine in NgAgo to create NgAgo (R40C) as described previously (25). Aspartic acids 663 and 738 were replaced with alanine to create NgAgoDM (D663A, D738A). Primers are listed in Table S2.

### Southern blotting.

Southern blotting was performed using a probe corresponding to the kanamycin gene, which was amplified by PCR, purified by agarose gel electrophoresis, sequenced, and labeled by digoxin (Fig. S1). The experiment was performed by GenScript, Inc. (Nanjing, China).

### Spot-plating assay.

Expression vectors for NgAgo or empty pET28a plasmid were transformed into E. coli, and 3 single colonies were picked from each plate and inoculated into LB liquid medium containing the corresponding antibiotics. Cells were grown in a shaking incubator at 37°C until the OD_600_ approached 0.5 (∼2.5 × 10^8^ cells per mL). Cells were diluted to 10^−2^, and then serial 100-fold dilutions were made until 10^−6^; 5 μL of each dilution was spotted on the LB plates supplemented with or without antibiotics and inducers as designated for specific experiments and placed in a thermostatic incubator for 24 h.

### Protein expression.

NgAgo protein samples were prepared using the same method described previously (25). Induction of expression from the T7 promoter, Lac promoter, or Tac promoter was induced by adding IPTG (0.5 mM) at 37°C for 4 h. Protein expressed by pBAD promoter was induced by adding arabinose (10 mM) at 37°C for 4 h.

### Western blotting.

Western blots were performed as described previously using His-tag primary antibody (66005-1-Ig; 1:10,000; Proteintech) and goat anti-mouse secondary antibody (CW0102S; 1:5,000; CWBIO) (25).

### BL21-pKD46 competent cell preparation.

Bacteriophage lambda RED recombination proteins encoded by plasmid pKD46 were introduced to enhance homologous sequence-directed recombination into BL21(DE3). This created strains with integrated NgAgo and enhanced green fluorescent protein expressed from the bacterial chromosome instead of a plasmid vector. The pKD46 plasmid was first transformed into BL21(DE3), and a single colony picked from LB+Amp plate was inoculated into 2 mL LB medium containing ampicillin at 30°C with overnight shaking. A volume of 2 mL of saturated cells was added into 200 mL LB medium supplemented with ampicillin and arabinose for induction. Bacterial cells were cultivated with shaking at 30°C for about 2 h until the OD_600_ reached 0.5. Cells were chilled on ice for more than 20 min and then collected by refrigerated centrifugation at 6,000 × *g* for 15 min. The cell pellet was washed with cold double-distilled water (ddH_2_O) once and with cold 10% (wt/vol) glycerol twice and then resuspended with 2.5 mL cold 10% (wt/vol) glycerol and divided into small portions with 100 μL per tube (1 × 10^8^ cells/tube) and stored at −80°C for future transformation.

### *NgAgo/EGFP* integrated BL21(DE3) strain construction and competent cell preparation.

Integration plasmid pB-LA-Kan-NgAgo-RA was digested with BamHI, and a 4,816-bp linear integration cassette was separated and purified by agarose gel electrophoresis. Integration plasmid pB-LA-Kan-EGFP-RA was digested with EcoRI and HindIII, and a 1,854-bp linear integration cassette was similarly purified.

Linear integration cassettes were electroporated into BL21(DE3)-pKD46 competent cells and allowed to recover in 1 mL LB medium supplemented with ampicillin and arabinose for 2 h at 30°C. Cells were collected by centrifugation, plated on LB+Kan+IPTG plates, and grown in a thermostatic incubator overnight. Colonies were picked for colony PCR to identify the integrated cells using primers genome-F and genome-R (Table S2), and the positive products were sent for sequencing. Colonies with correctly integrated cassettes were cultivated in 2 mL LB medium supplemented with kanamycin at 37°C overnight. Glycerol was added to a final concentration of 30% (wt/vol), and samples were frozen at −80°C.

Frozen BL21::NgAgo cells were streaked on LB+Kan plates and single colonies inoculated into 2 mL LB medium containing kanamycin at 37°C overnight. A volume of 2 mL of saturated cells was added into 200 mL LB medium supplemented with kanamycin. Bacterial cells were shaken at 37°C for about 2 h until the OD_600_ reached 0.5. Cells were chilled on ice for at least 20 min and collected by centrifugation. Cell pellets were washed with cold ddH_2_O once and cold 10% (wt/vol) glycerol twice, resuspended with 2.5 mL cold 10% (wt/vol) glycerol, divided into small portions with 100 μL per tube (1 × 10^8^ cells/tube), and stored at −80°C.

### Plasmid maintenance assay.

pET32a, pUC57, and pUC57-araC were transformed into BL21::NgAgo and BL21(DE3) to compare the effect of NgAgo expression on plasmid maintenance. A total of 3 colonies in each transformation group were picked and inoculated into 2 mL LB+Amp+kan and LB+Amp medium and cultivated at 37°C until the OD_600_ reached 0.5. The cell culture was supplemented with glycerol to a final concentration of 25% (wt/vol) and then stored at −80°C overnight. The frozen cell culture was thawed on ice and inoculated into liquid LB with the addition of IPTG (0.2 mM) and antibiotic corresponding to the plasmids and bacterial strains and cultured for 12 h. Aliquots of BL21::NgAgo transformation cell culture were plated on LB+Kan and LB+Kan+Amp plates, BL21(DE3) transformation cell cultures were plated on LB and LB+Amp plates, and this was termed passage 1 (P1). The plasmid-free cell number was calculated as (1 − [colony number on LB+Amp plates/colony number on LB plates]) × 100%. The cell culture was grown at 37°C and passaged the same as passage 1 every 12 h. P1, P2, and P4/P7 were calculated and analyzed by SPSS.

### T7 phage stock solution preparation.

A single colony of BL21(DE3) cells was inoculated into 2 mL LB liquid medium until the OD_600_ reached 0.5. Aliquots of frozen bacteriophage T7 (ATCC BAA-1025-B2) were thawed on ice and added to cell cultures, which were grown until the suspension became transparent. The cell culture was extracted with an equal volume of chloroform, and the mixture was centrifuged at 12,000 rpm at 4°C for 10 min after vortexing. The upper supernatant was transferred into a new tube and kept at 4°C for no more than a week.

### Analysis of bacteriophage T7 infection.

NgAgo and NgAgo mutant expression vectors were transformed into BL21(DE3) cells respectively, and a single colony from each group was inoculated into 2 mL LB liquid medium for overnight culture. Vector-free BL21(DE3) was cultured as the control. A volume of 0.5 mL cell culture was inoculated into 10 mL fresh LB liquid medium and cultured until the OD_600_ reached 0.5. IPTG (0.5 mM) or arabinose (10 mM) was added to NgAgo and NgAgo mutant transformation groups, and cells were cultured for 2 h to allow protein expression. After 2 h, OD_600_ was measured, and cells at an OD_600_ of 0.5 from each group was taken and mixed with 100 μL T7 phage stock and incubated at 37°C for 30 min. The phage-infected cell culture was added into 5 mL 0.8% upper solid LB medium, and the mixture was gently mixed well and was poured on the LB plates. Phage plaques were visible about 4 h postinfection when plates were incubated at 37°C.

### Bacteriophage T7 genomic DNA extraction and identification.

T7 genomic DNA was extracted as described previously: https://www.researchgate.net/post/What_is_the_best_method_for_the_isolation_of_bacteriophage_DNA. A volume of 200 mL of T7 phage-infected cell culture was centrifuged to collect the cell pellet. Cell pellet was resuspended with 20 mL SM buffer (100 mM NaCl, 25 mM Tris-HCl, pH 7.5, 8 mM MgSO_4_, 0.01% [wt/vol] gelatin), treated with 10 μL RNase A (5 mg/mL) and 10 μL DNase I (10 mg/mL), and incubated at 37°C overnight. After incubation, 8 mL precipitant solution (33% polyethylene glycol 8000, 3.3 M NaCl) was added into the mixture and gently mixed well and stored at −80°C for 1 h. The mixture was centrifuged at 12,000 rpm at 4°C for 45 min, and the supernatant was removed. We resuspended the cell pellet with 5 mL SM buffer and centrifuged it at 12,000 rpm at 4°C for 5 min. We collected and resuspended the cell pellet with 600 μL SM buffer in an Eppendorf tube, and two equal volumes of phenol-chloroform-isoamyl alcohol (25:24:1) were added into the tube. The mixture was thoroughly mixed and centrifuged at 12,000 rpm for 10 min. The upper aqueous layer was transferred into a new tube, and an equal volume of chloroform was added and mixed well and was centrifuged at 12,000 rpm at 4°C for 20 min. The upper aqueous layer was transferred into a new tube, 0.3 M sodium acetate (pH 5.3) was added into the tube, and equal volume of isopropyl alcohol was added. The tube was incubated at −80°C for 10 min after vortexing. The tube was centrifuged at 12,000 rpm at 4°C for 20 min and the supernatant was removed. A volume of 700 μL cold, 70% ethanol was added and then centrifuged at 12,000 rpm at 4°C for 10 min. The dried pellet was dissolved in 50 μL TE buffer (10 mM Tris-HCl and 1 mM EDTA, pH 8.0).

A volume of 100 ng genomic DNA was used as the template to perform PCR with primers T7p17-F/R (listed in Table S2), the annealing temperature was 58°C, and the amplification product was 699 bp in molecular size.

## References

[1] Koonin EV. 2017. Evolution of RNA- and DNA-guided antivirus defense systems in prokaryotes and eukaryotes: common ancestry vs convergence. Biol Direct 12:5. doi:10.1186/s13062-017-0177-2. 28187792 PMC5303251

[2] Swarts DC, Makarova K, Wang Y, Nakanishi K, Ketting RF, Koonin EV, Patel DJ, van der Oost J. 2014. The evolutionary journey of Argonaute proteins. Nat Struct Mol Biol 21:743–753. doi:10.1038/nsmb.2879. 25192263 PMC4691850

[3] Makarova KS, Wolf YI, van der Oost J, Koonin EV. 2009. Prokaryotic homologs of Argonaute proteins are predicted to function as key components of a novel system of defense against mobile genetic elements. Biol Direct 4:29. doi:10.1186/1745-6150-4-29. 19706170 PMC2743648

[4] Bohmert K, Camus I, Bellini C, Bouchez D, Caboche M, Benning C. 1998. AGO1 defines a novel locus of Arabidopsis controlling leaf development. EMBO J 17:170–180. doi:10.1093/emboj/17.1.170. 9427751 PMC1170368

[5] Ketting RF. 2011. The many faces of RNAi. Dev Cell 20:148–161. doi:10.1016/j.devcel.2011.01.012. 21316584

[6] Swarts DC, Jore MM, Westra ER, Zhu Y, Janssen JH, Snijders AP, Wang Y, Patel DJ, Berenguer J, Brouns SJJ, van der Oost J. 2014. DNA-guided DNA interference by a prokaryotic Argonaute. Nature 507:258–261. doi:10.1038/nature12971. 24531762 PMC4697943

[7] Swarts DC, Hegge JW, Hinojo I, Shiimori M, Ellis MA, Dumrongkulraksa J, Terns RM, Terns MP, van der Oost J. 2015. Argonaute of the archaeon Pyrococcus furiosus is a DNA-guided nuclease that targets cognate DNA. Nucleic Acids Res 43:5120–5129. doi:10.1093/nar/gkv415. 25925567 PMC4446448

[8] Cao Y, Sun W, Wang J, Sheng G, Xiang G, Zhang T, Shi W, Li C, Wang Y, Zhao F, Wang H. 2019. Argonaute proteins from human gastrointestinal bacteria catalyze DNA-guided cleavage of single- and double-stranded DNA at 37 degrees C. Cell Discov 5:38. doi:10.1038/s41421-019-0105-y. 31636952 PMC6796838

[9] Hegge JW, Swarts DC, Chandradoss SD, Cui TJ, Kneppers J, Jinek M, Joo C, van der Oost J. 2019. DNA-guided DNA cleavage at moderate temperatures by Clostridium butyricum Argonaute. Nucleic Acids Res 47:5809–5821. doi:10.1093/nar/gkz306. 31069393 PMC6582352

[10] Kuzmenko A, Oguienko A, Esyunina D, Yudin D, Petrova M, Kudinova A, Maslova O, Ninova M, Ryazansky S, Leach D, Aravin AA, Kulbachinskiy A. 2020. DNA targeting and interference by a bacterial Argonaute nuclease. Nature 587:632–637. doi:10.1038/s41586-020-2605-1. 32731256

[11] Zander A, Willkomm S, Ofer S, van Wolferen M, Egert L, Buchmeier S, Stöckl S, Tinnefeld P, Schneider S, Klingl A, Albers S-V, Werner F, Grohmann D. 2017. Guide-independent DNA cleavage by archaeal Argonaute from Methanocaldococcus jannaschii. Nat Microbiol 2:17034. doi:10.1038/nmicrobiol.2017.34. 28319081 PMC7616673

[12] Liu Y, Li W, Jiang X, Wang Y, Zhang Z, Liu Q, He R, Chen Q, Yang J, Wang L, Wang F, Ma L. 2021. A programmable omnipotent Argonaute nuclease from mesophilic bacteria Kurthia massiliensis. Nucleic Acids Res 49:1597–1608. doi:10.1093/nar/gkaa1278. 33444443 PMC7897485

[13] Kaya E, Doxzen KW, Knoll KR, Wilson RC, Strutt SC, Kranzusch PJ, Doudna JA. 2016. A bacterial Argonaute with noncanonical guide RNA specificity. Proc Natl Acad Sci USA 113:4057–4062. doi:10.1073/pnas.1524385113. 27035975 PMC4839417

[14] Miyoshi T, Ito K, Murakami R, Uchiumi T. 2016. Structural basis for the recognition of guide RNA and target DNA heteroduplex by Argonaute. Nat Commun 7:11846. doi:10.1038/ncomms11846. 27325485 PMC4919518

[15] Swarts DC, Szczepaniak M, Sheng G, Chandradoss SD, Zhu Y, Timmers EM, Zhang Y, Zhao H, Lou J, Wang Y, Joo C, van der Oost J. 2017. Autonomous generation and loading of DNA guides by bacterial Argonaute. Mol Cell 65:985–998. doi:10.1016/j.molcel.2017.01.033. 28262506 PMC5779613

[16] Wang Y, Juranek S, Li H, Sheng G, Tuschl T, Patel DJ. 2008. Structure of an argonaute silencing complex with a seed-containing guide DNA and target RNA duplex. Nature 456:921–926. doi:10.1038/nature07666. 19092929 PMC2765400

[17] Garcia-Quintans N, Bowden L, Berenguer J, Mencia M. 2019. DNA interference by a mesophilic Argonaute protein, CbcAgo. F1000Res 8:321. doi:10.12688/f1000research.18445.2. 32055395 PMC6961421

[18] Kuzmenko A, Yudin D, Ryazansky S, Kulbachinskiy A, Aravin AA. 2019. Programmable DNA cleavage by Ago nucleases from mesophilic bacteria Clostridium butyricum and Limnothrix rosea. Nucleic Acids Res 47:5822–5836. doi:10.1093/nar/gkz379. 31114878 PMC6582412

[19] Lisitskaya L, Aravin AA, Kulbachinskiy A. 2018. DNA interference and beyond: structure and functions of prokaryotic Argonaute proteins. Nat Commun 9:5165. doi:10.1038/s41467-018-07449-7. 30514832 PMC6279821

[20] Swarts DC, Koehorst JJ, Westra ER, Schaap PJ, van der Oost J. 2015. Effects of Argonaute on gene expression in Thermus thermophilus. PLoS One 10:e0124880. doi:10.1371/journal.pone.0124880. 25902012 PMC4406477

[21] Jolly SM, Gainetdinov I, Jouravleva K, Zhang H, Strittmatter L, Bailey SM, Hendricks GM, Dhabaria A, Ueberheide B, Zamore PD. 2020. Thermus thermophilus Argonaute functions in the completion of DNA replication. Cell 182:1545–1559. doi:10.1016/j.cell.2020.07.036. 32846159 PMC7502556

[22] Qi J, Dong Z, Shi Y, Wang X, Qin Y, Wang Y, Liu D. 2016. NgAgo-based fabp11a gene knockdown causes eye developmental defects in zebrafish. Cell Res 26:1349–1352. doi:10.1038/cr.2016.134. 27834346 PMC5143420

[23] Wu Z, Tan S, Xu L, Gao L, Zhu H, Ma C, Liang X. 2017. NgAgo-gDNA system efficiently suppresses hepatitis B virus replication through accelerating decay of pregenomic RNA. Antiviral Res 145:20–23. doi:10.1016/j.antiviral.2017.07.005. 28709658

[24] Olovnikov I, Chan K, Sachidanandam R, Newman DK, Aravin AA. 2013. Bacterial argonaute samples the transcriptome to identify foreign DNA. Mol Cell 51:594–605. doi:10.1016/j.molcel.2013.08.014. 24034694 PMC3809076

[25] Xing J, Ma L, Cheng X, Ma J, Wang R, Xu K, Mymryk JS, Zhang Z. 2021. Expression and functional analysis of the Argonaute protein of Thermus thermophilus (TtAgo) in E. coli BL21(DE3). Biomolecules 11:524. doi:10.3390/biom11040524. 33807395 PMC8067300

[26] Masukata H, Tomizawa J. 1986. Control of primer formation for ColE1 plasmid replication: conformational change of the primer transcript. Cell 44:125–136. doi:10.1016/0092-8674(86)90491-5. 2416472

[27] Seo JH, Baek SW, Lee J, Park JB. 2017. Engineering Escherichia coli BL21 genome to improve the heptanoic acid tolerance by using CRISPR-Cas9 system. Biotechnol Bioproc E 22:231–238. doi:10.1007/s12257-017-0158-4.

[28] Willkomm S, Makarova KS, Grohmann D. 2018. DNA silencing by prokaryotic Argonaute proteins adds a new layer of defense against invading nucleic acids. FEMS Microbiol Rev 42:376–387. doi:10.1093/femsre/fuy010. 29579258 PMC5995195

[29] Rocha EP, Danchin A. 2002. Base composition bias might result from competition for metabolic resources. Trends Genet 18:291–294. doi:10.1016/S0168-9525(02)02690-2. 12044357

[30] Golomb M, Chamberlin M. 1974. Characterization of T7-specific ribonucleic acid polymerase. IV. Resolution of the major in vitro transcripts by gel electrophoresis. J Biol Chem 249:2858–2863. doi:10.1016/S0021-9258(19)42709-9. 4828324

