# Supplementary figures and images for: Prokaryotic Argonaute Protein from Natronobacterium gregoryi Requires RNAs To Activate for DNA Interference In Vivo
Source: mBio. 2022 Mar 28;13(2):e03656-21. doi: 10.1128/mbio.03656-21 (PMC9040764; doi:10.1128/mbio.03656-21)

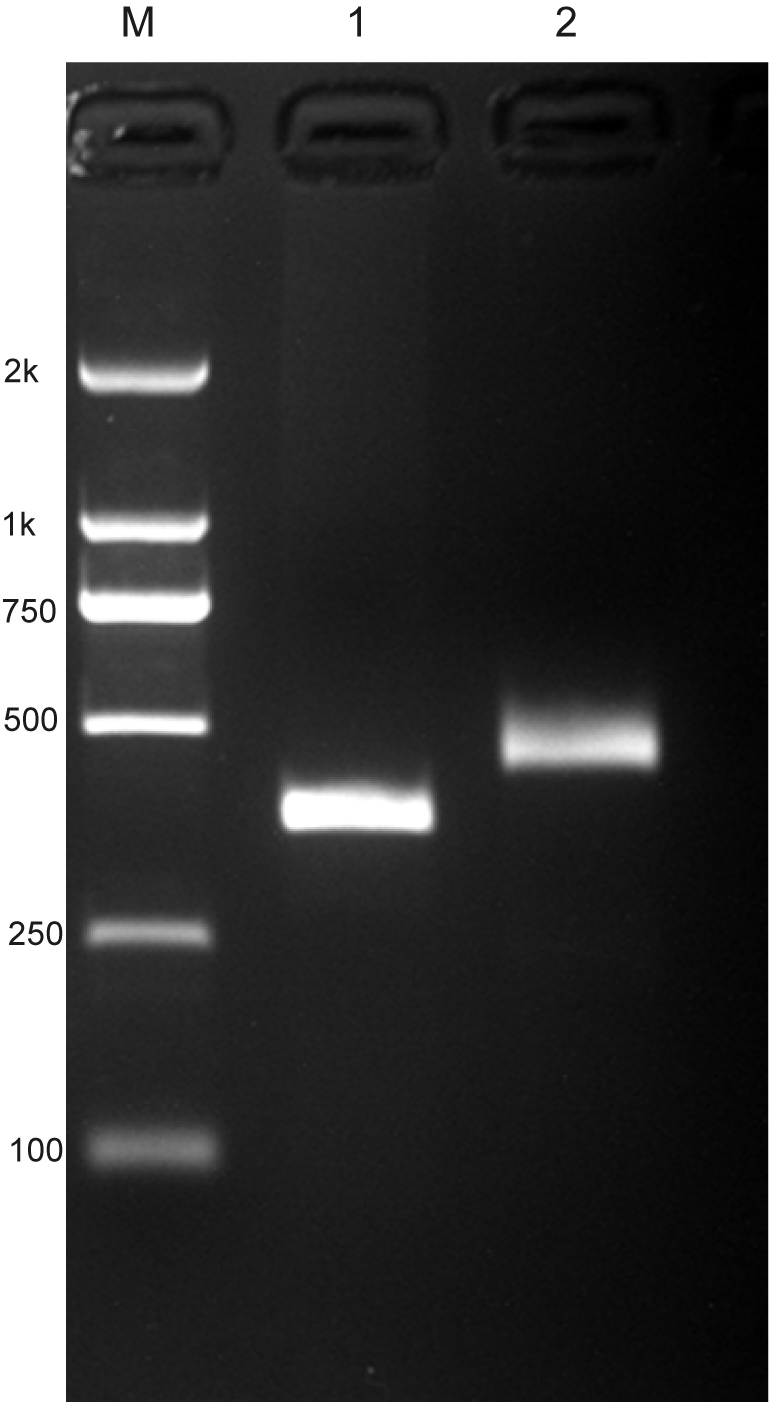

Supplement: FIG S1 [file mbio.03656-21-sf001.tif]

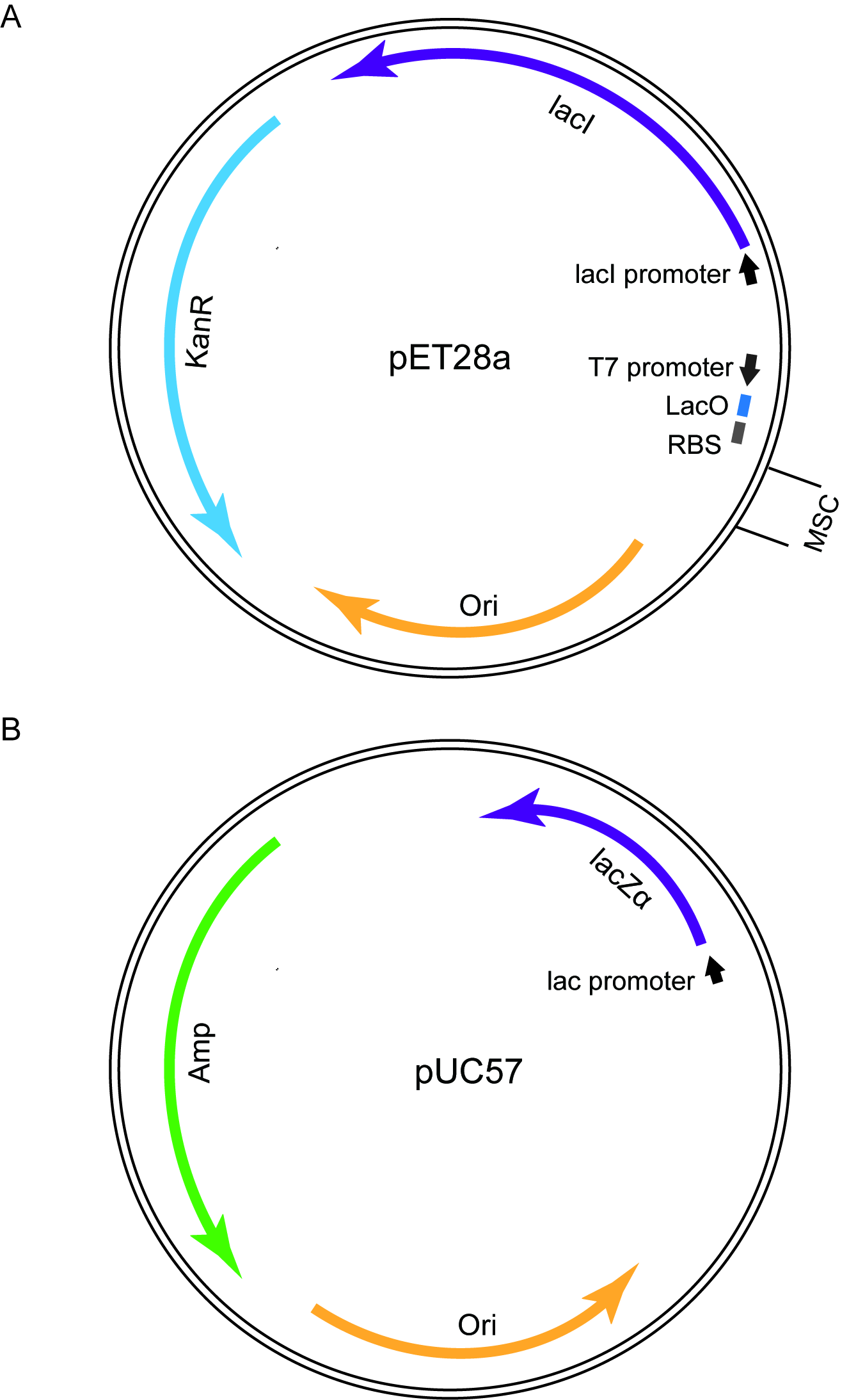

Supplement: FIG S2 [file mbio.03656-21-sf002.tif]

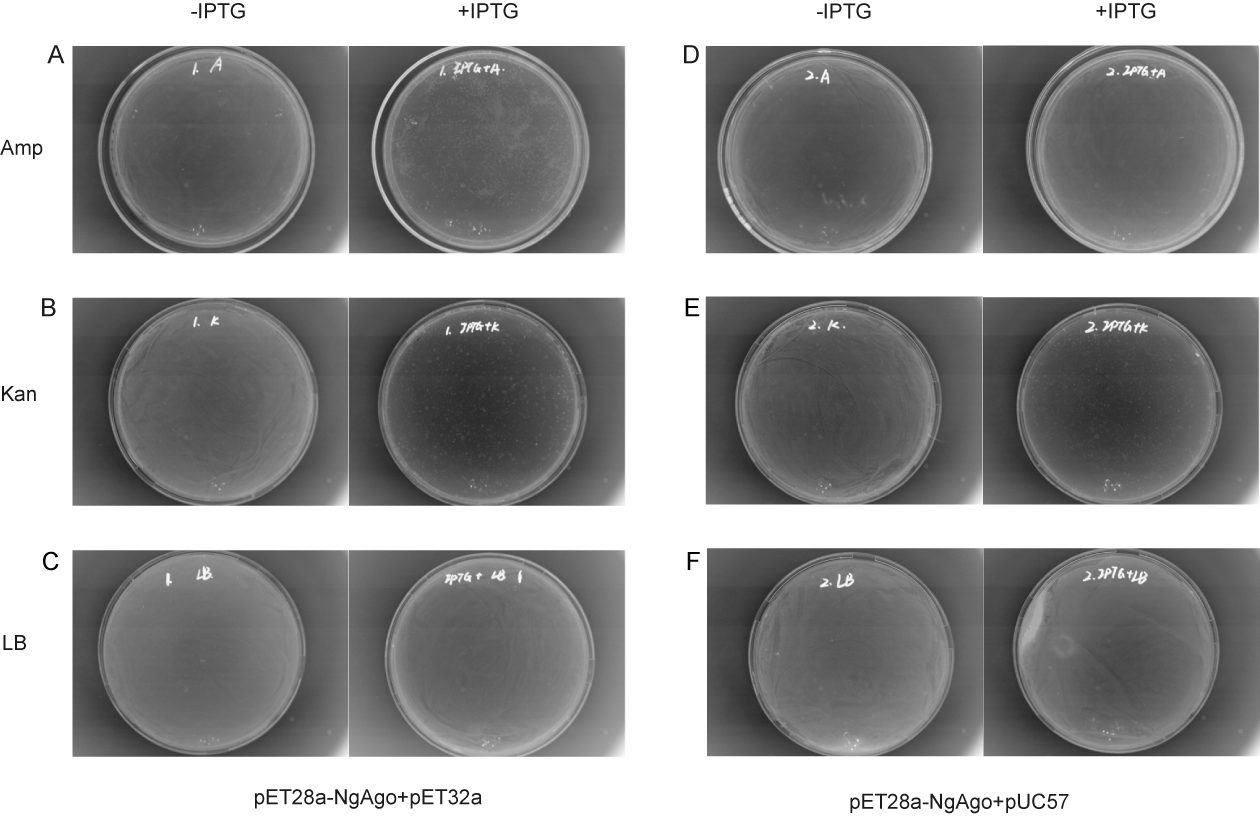

Supplement: FIG S3 [file mbio.03656-21-sf003.tif]

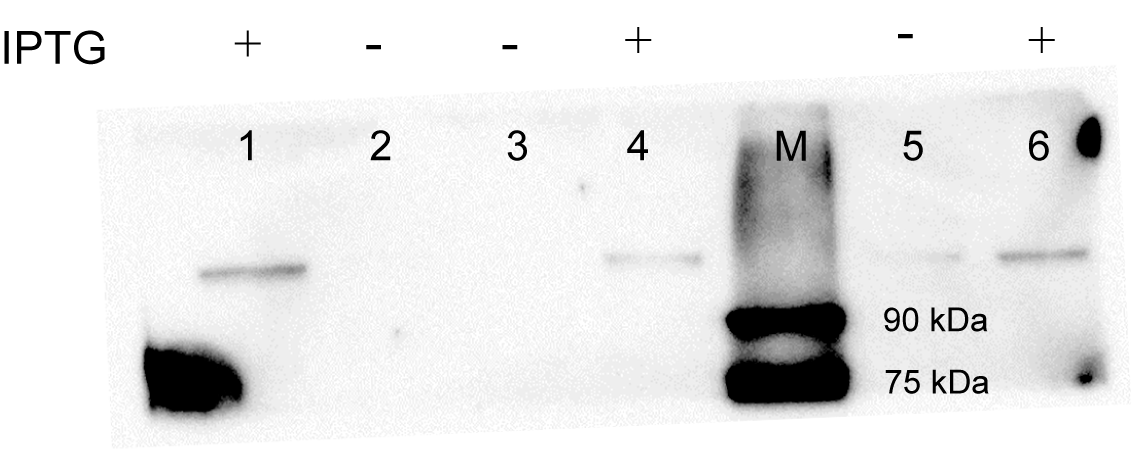

Supplement: FIG S4 [file mbio.03656-21-sf004.tif]

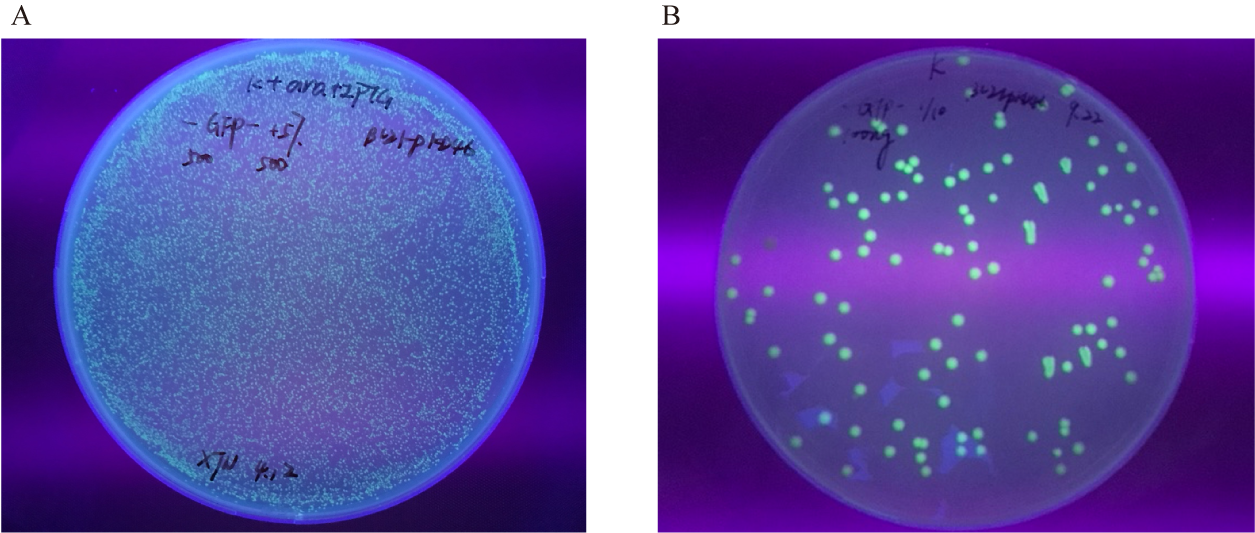

Supplement: FIG S5 [file mbio.03656-21-sf005.tif]

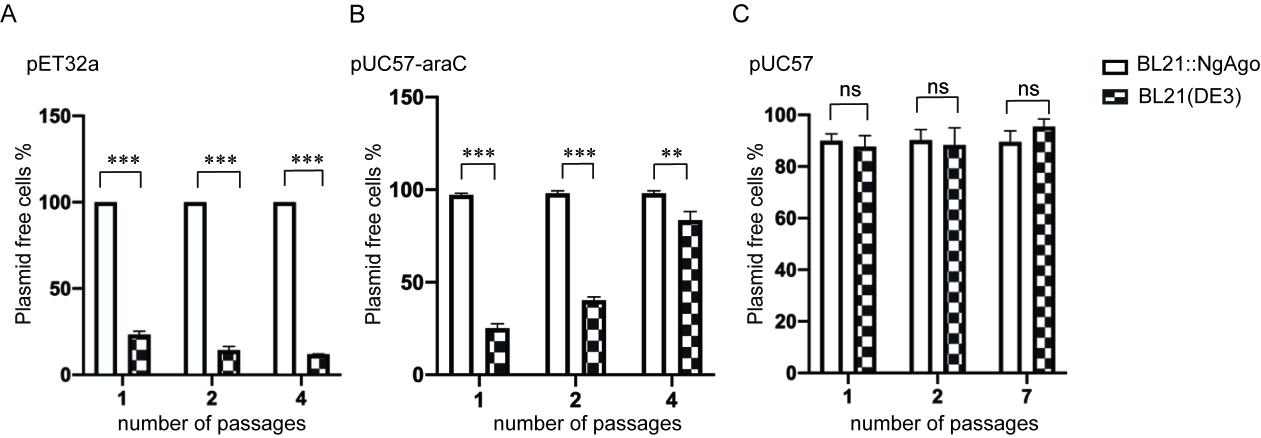

Supplement: FIG S6 [file mbio.03656-21-sf006.tif]
